# Natural selection of mitochondria during somatic lifetime promotes healthy aging

**DOI:** 10.3389/fnene.2013.00007

**Published:** 2013-08-12

**Authors:** Anders Rodell, Lene J. Rasmussen, Linda H. Bergersen, Keshav K. Singh, Albert Gjedde

**Affiliations:** ^1^Department of Nuclear Medicine & PET Centre, Aarhus University HospitalAarhus, Denmark; ^2^Center for Healthy Aging and Department of Cellular and Molecular Medicine, University of CopenhagenCopenhagen, Denmark; ^3^Department of Neuroscience and Pharmacology and Center for Healthy Aging and, University of CopenhagenCopenhagen, Denmark; ^4^The Brain and Muscle Energy Group, Centre for Molecular Biology and Neuroscience and Institute for Basic Medical Sciences, University of OsloOslo, Norway; ^5^Departments of Genetics, Pathology and Environmental Health and UAB Comprehensive Cancer Center, University of Alabama at BirminghamBirmingham, AL, USA; ^6^Center for Free Radical Biology, University of Alabama at BirminghamBirmingham, AL, USA; ^7^Center of Functionally Integrative Neuroscience, Aarhus UniversityAarhus, Denmark; ^8^McConnell Brain Imaging Center, Montreal Neurological Institute, McGill UniversityMontreal, Quebec, QC, Canada; ^9^Department of Radiology and Radiological Science, Johns Hopkins UniversityBaltimore, Maryland, MD, USA

**Keywords:** energy metabolism, epigenetics, evolutionary bottleneck, mitochondrial adaptation, mitochondrial maladaptation

## Abstract

Stimulation of mitochondrial biogenesis during life-time challenges both eliminates disadvantageous properties and drives adaptive selection of advantageous phenotypic variations. Intermittent fission and fusion of mitochondria provide specific targets for health promotion by brief temporal stressors, interspersed with periods of recovery and biogenesis. For mitochondria, the mechanisms of selection, variability, and heritability, are complicated by interaction of two independent genomes, including the multiple copies of DNA in each mitochondrion, as well as the shared nuclear genome of each cell. The mechanisms of stress-induced fission, followed by recovery-induced fusion and biogenesis, drive the improvement of mitochondrial functions, not only as directed by genotypic variations, but also as enabled by phenotypic diversity. Selective adaptation may explain unresolved aspects of aging, including the health effects of exercise, hypoxic and poisonous preconditioning, and tissue-specific mitochondrial differences. We propose that intermittent purposeful enhancement of mitochondrial biogenesis by stressful episodes with subsequent recovery paradoxically promotes adaptive mitochondrial health and continued healthy aging.

## Maladaptive variability vs. adaptive specialization

Convention holds that mitochondrial gene variability (heteroplasmy) is detrimental to organisms (Elliott et al., 2008), because heteroplasmy alone leads to unexpected genetic and behavioral instabilities, even when variants of mtDNA appear to perform well with unchanged nuclear support (Lane, 2012; Sharpley et al., 2012). The continued performance has been the reason for the general claim that mtDNA molecules are identical at birth in the vast majority of humans (homoplasmy) (Taylor and Turnbull, 2005). Homoplasmy is maintained by the asexual maternal inheritance of eukaryotes (Giles et al., 1980). Recent increases of the resolution of detection of mtDNA variability revealed that low level heteroplasmy is universal in human mtDNA (Payne et al., 2013), and some variants expand clonally to cause disease at old age (Elliott et al., 2008).

Here, we present the contrasting view that the effects of mitochondrial variability, in a broader sense, are not limited to a decline from a healthy norm, leading to unhealthy aging and disease, but may serve also as the fabric of positive adaptive responses, at the genetic and epigenetic levels, to challenging bioenergetic events. The roles of mitochondrial biogenesis and dynamic fission and fusion mechanisms are vital to the maintenance of healthy mitochondrial populations, and impairment of the respective mechanisms is implicated in many age-related diseases. Twig and Shirihai (2011) and Kowald and Kirkwood (2011a,b) recently convincingly argued that mitochondrial fission and fusion together provide a mechanism of elimination of mtDNA with damages that limit the efficiency of respiration (Twig and Shirihai, 2011). However, from the perspective of the unfolding of an advantageous potential, these mechanisms have novel and wide importance to the understanding of the consequences of stress-induced fission, followed by recovery-induced fusion and mitochondrial biogenesis.

We posit that natural selection not only serves to adapt mitochondria to different tissue requirements during development (Kuznetsov and Margreiter, 2009), but the mechanism described as mitocheckpoint (Minocherhomji et al., 2012) also is exploited to rejuvenate the mitochondrial population during aging in order to maintain the respiratory capacity required for continued healthy aging. Recently, Jose et al. (2013) reviewed the adaptive biology of mitoplasticity as a protective mechanism against aging, diabetes, cancer and neurodegenerative diseases, which we here extend to the specific and directed promotion of healthy aging.

## Multiple outcomes of selective challenges

Natural selection of variations in its original paleo-Darwinian formulation is opposed to the uniquely genetic focus of neo-Darwinism. As cellular organelles, mitochondria are unique in animal cells as carriers of individual genomes, interacting through transcription factors with the common nuclear genome (nDNA). Because of the interaction, mitochondria are subject to complex selection, ranging from genetic selection of heteroplasmic variants to the epigenetic environment in which the mitochondria pass through multiple generational cycles. Epigenetics allows a cell or network to store the effects of experiences and modify the decoding of the genome. Depending on the unit, epigenetic memories are stored as methylation, altered microRNA profiles, nucleosome positions, or chromatin alterations in the case of nDNA. Epigenetic imprints can be transferred to offspring units and passed to subsequent generations in eukaryotes (Grossniklaus et al., 2013) and prokaryotes (Adam et al., 2008; Ni et al., 2012). Importantly, the epigenetic environment involves both nuclear and mitochondrial transcriptions (Minocherhomji et al., 2012), in principle creating a high potential for the phenotypical variability targeted by selection.

Notable examples of somatic selection and specialization within the somatic lifetime of cellular units include the antibody-selective proliferation of adapted units of the immune system in response to infections (Jerne, 1955), and the pruning of cerebral cortex connections after childhood (Edelman and Mountcastle, 1978; Edelman and Reeke, 1982). Indeed, for mitochondria, some embryogenic specialization happens, such that mitochondria vary phenotypically in different tissues (Kuznetsov and Margreiter, 2009). Negative selection effectively eliminates damaged mitochondria by autophagy (Feng et al., 2013), and the role of mitochondria in apoptosis can be interpreted as a response to selection pressure imposed by abnormally active free radical leakage (Hengartner, 1998; Blackstone and Green, 1999).

From an evolutionary point of view, we argue that an absent positive selection of a replicatively advantageous phenotypical response to a challenge would constitute an asymmetry, also among healthy mitochondria. In order for any selective adaptation to persist from one mitochondrial generation to the next, when cloned, the adaptation must be genetically or epigenetically extant and be continuously transferable to the new clones, or a change of the cellular environment that favors the specific selective advantage must persist.

## Selection by aging

Metabolic stress is a well-characterized stimulus for increased mitochondrial mass in skeletal muscle, particularly through the AMPK/PGC-1a-dependent signaling (Gurd et al., 2011). However, it is equally well-established that increased metabolism affects redox regulation (Chau et al., 2010). Oxidative stress has been held to signal mitochondrial biogenesis (Davies et al., 1982), but free radical leakage as well has been linked to aging, depending on threshold, based on clear demonstration of extensive oxidative damage as function of age in the last quarter of life (Radak et al., 2013).

Aging is associated with decreasing bio-energetic capacity, as mitochondria increasingly become unable to meet the respiratory energy demands of cells (Dresler et al., 2012). Free radicals increasingly damage mtDNA with age, leading to an age-dependent state of variable heteroplasmy in individual cells, with detrimental effects on the bio-energetic capacity of the tissue (He et al., 2010). Studies of mtDNA mutator mice show that increased accumulation of damaged mtDNA exacerbates the heteroplasmic conversion, with rapid onset of unstable health as the hallmark of aging and onset of age-related diseases. Such heteroplasmic variation can be described as the consequence of an age-dependent lack of selection (Dai et al., 2013).

In the brain, effects of aging on mitochondrial health are particularly important, as neuronal function crucially depends on sufficiently high rates of oxidative phosphorylation (Bolaños et al., 2009), mediated by mitochondria moving for long distances, and on neurons that persist without replacement from stem cells with healthy mitochondria.

## Selection by brain activation

Cerebral energy metabolism is covered almost exclusively by oxidation of glucose with a molar ratio between the net uptakes of oxygen and glucose, the oxygen-glucose index (OGI), close to 6 (Quistorff et al., 2008). Upon activation, the relative changes of oxygen and glucose uptakes appear to diverge from the steady-state norm, resulting in decline of the OGI to values as low as 2.8 (Seifert et al., 2009). The increases of blood flow and glucose consumption are in contrast to the comparatively unchanged oxygen consumption. The benefit of this mismatch is uncertain, because of the absent flow-limitation of glucose delivery and the substantial flow-limitation of oxygen delivery (Gjedde et al., 2005).

The mismatch is followed by significant increase of the tissue lactate concentration, which may be a signal for increase of blood flow (Bergersen and Gjedde, 2012). The absence of mitochondria at the post-synaptic density of dendritic spines of neurons may explain the mismatch at the onset of stimulation, followed by return to the resting state average, with gradual accumulation of mitochondria at the spines upon continued stimulation (Li et al., 2004).

We claim that the mismatch can serve to stimulate the selection of mitochondria with properties that are advantageous to the continued stimulation and the proliferation of dendritic spines, when mitochondria accumulate at certain types of dendritic spines, and the OGI again reaches the steady-state norm close to 5.5 (Rasmussen et al., 2010a,b; Vafaee et al., 2012). The recent discovery of the lactate receptor at the post synaptic density (Lauritzen et al., 2013), where also monocarboxylate transporter 2 is localized (Bergersen et al., 2001), suggests that lactate signaling could be involved in the regulation of mitochondrial dynamics at spines. Alternatively, the lactate signals may also reflect differences between changes of the oxidative and glycolytic pathways that exist because it is suboptimal to run the brain at full efficiency at all times.

Starvation and ketogenic dieting lowers the brain's glucose consumption in association with the rise of ketone bodies in the circulation and induction of sufficient transport capacity of the blood-brain barrier (Gjedde and Crone, 1975; Zhang et al., 2013). The change of metabolism lowers mitochondrial susceptibility to stress, because the ketone bodies sidestep complex I of the ETC (Kang et al., 2013).

## Phenotypic variation

Natural life-time selection of mitochondria requires variability that either persists through clonal expansion or persistently regulates the epigenetic environment of mitochondria. The possible forms include:

### Mitochondrial DNA variation

Sexual reproduction and oocyte selection (Jansen and de Boer, 1998) together prepare a homeoplasmic population of mitochondria for maternal inheritance to offspring and establish that the mitochondrial genome functions well against the background of the nuclear genome (Lane, 2012). Heteroplasmy increases at old age, giving rise to variation of the mtDNA. This heteroplasmy may compromise the interaction with the nuclear DNA, to the detriment of respiratory capacity (Minocherhomji et al., 2012).

### Tissue variation

Mitochondria exhibit tissue specific differences (Kuznetsov and Margreiter, 2009). In rats, liver mitochondria are the most thermo-dynamically efficient at ATP production using oxidative phosphorylation. Heart and brain mitochondrial systems utilize more oxygen, but can produce ATP at a faster rate than liver tissue (Cairns et al., 1998).

### Copy number

An iso-genetic population of mitochondria may differ with respect to the number of mtDNA copies. As the copies are linked to ETC complexes (Kowald and Kirkwood, 2011a,b), the differences may induce variations of respiratory capacity.

### Transcription factors

Variations in transcription factors possibly reflect other variations, genotypical or epigenetic, but are known to regulate respiratory capacity both in short and long terms, as reviewed recently by Dresler et al. (2012).

### Epigenetically inherited adaptations

In bacteria without DNA mutation or plasmid uptake, epigenetically inherited adaptation can be the cause of variability (Adam et al., 2008; Ni et al., 2012; Grossniklaus et al., 2013), and is inherited, although the bacteria also easily revert. As the mitochondria are believed to be of bacterial origin, it is probable that phenotypical variation can undergo selective inheritance without an underlying genotypical diversity of the respective mitochondria, as in bacteria. However, it should be noted that epigenetic modifications could reside on both nuclear and mitochondrial genomes (Enríquez et al., 1999) and as such could have a more persistent effect than in bacteria.

These variations have the potential to be disruptive of efficient respiration, as well as to provide a basis for adaptation by selection.

## Selective mechanisms

Although much remains to be learned about the complex mechanisms of biogenesis and turnover of mitochondria during aging (Jose et al., 2013), the mechanisms are believed to be regulated by interplay with the nuclear genome, involving hormonal regulators, transcriptional factors and co-activators, sirtuins, and the fusion-fission cycles of mitochondria (Lopez-Lluch et al., 2008). Unsurprisingly, the regulatory mechanisms appear to be linked intimately to the balance of the organism's energy requirements and supply. The benefits of the fission and fusion steps of mitochondria are largely unknown, although these mechanisms regulate the number of mitochondria as well as their motility (Li et al., 2004; Knott and Bossy-Wetzel, 2008; DuBoff et al., 2013). Kowald and Kirkwood (2011a,b) hypothesize a unified framework that determines both how fission and fusion are regulated and how they maintain mitochondrial health. Recent findings suggest that mitochondria regularly fuse and join dynamic networks within which they exchange proteins, mtDNA, and lipids (Duvezin-Caubet et al., 2006; Knott and Bossy-Wetzel, 2008; Twig et al., 2008; Twig and Shirihai, 2011).

When different mtDNA copies coexist inside a single mitochondrion, or if mtDNA is shared in a fused network, the unit of genetic selection may be established by the speed of replication, rather than by the speed of mitochondrial reproduction. Therefore, it is possible that some forms of selection of mtDNA, limited by replication of DNA, happen to mitochondria of the network, the so called “survival of the tiny mtDNA” hypothesis (Kowald et al., 2005). The mechanism may account for the presence of single point mutations undergoing clonal expansion in individual aged cells.

Fusion and networking of mitochondria are held to increase the coordination between the needs of individual mitochondria in different parts of the cell and the transcriptional ability of the single nucleus to meet diverse demands of different mitochondria (Kowald and Kirkwood, 2011b; Twig and Shirihai, 2011). According to this hypothesis, fission, in turn, serves to expose mtDNA mutations by segregating the mtDNA into separate mitochondria with lower copy number per mitochondrion. Fission also serves to increase mitochondrial motility (DuBoff et al., 2013). The sequestration is possible only to the extent that the mutations are linked phenotypically to the inner mitochondrial membrane and therefore are detectable by the cell, for example in the form of ineffective respiratory capacity of the organelle (Kowald and Kirkwood, 2011a,b). The most likely signal for this detection is the increased ROS production by damaged mitochondria. Disruption of the carefully orchestrated fission and fusion balance potentially is a cause of mitochondrial dysfunction, and uncontrolled fission with mitochondrial fragmentation frequently is the result of a cellular insult and is turned on by oxidative and nitrosative stress, DNA damage, and elevated glucose levels, among other factors (Knott and Bossy-Wetzel, 2008).

**Figure 1:**
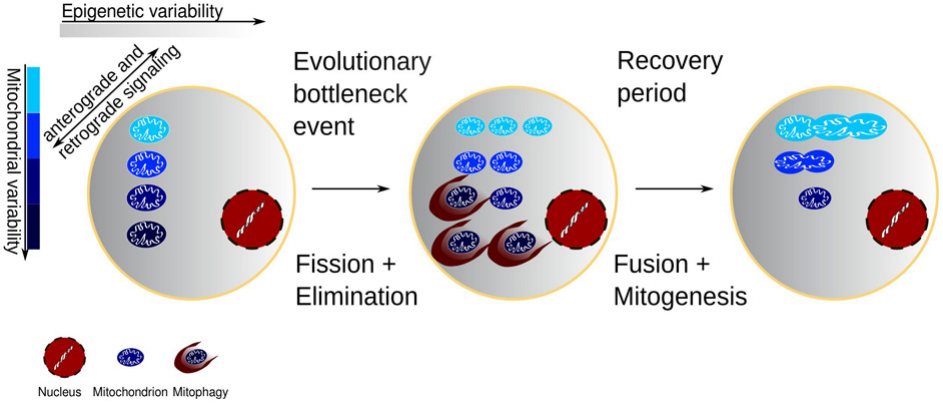
**Cell with variability of both mitochondrial function and the epigenetic environment of the mitochondria.** The mitochondria change the epigenetic environment through retrograde redox signaling, and conversely the epigenetic environment may favor some mitochondria. Environmental or chemotherapeutic challenges may expose the damaged mitochondrial segments and after a subsequent recovery the clonal expansion of the mitochondrial mass would come from a healthier population.

In perspective, the sequential mechanisms of fission, fusion, and biogenesis provide precisely what is needed for selection to adapt the mitochondrial population to the changing cellular environment throughout life, in response to intermittent exposure to environmental challenges and subsequent recovery. As illustrated in Figure 1, fission effectively creates a genetic bottleneck by allowing only the best adapted mitochondria to survive an insult or environmental change, at the same time using increased ROS levels to signal the need for compensatory mitochondrial biogenesis of a magnitude sufficient to match the capacity to the demand. The consequent biogenesis subserves the selection of surviving mitochondria by means of clonal expansion. The fusion and networking restore the coordination between the diversity of mitochondrial needs and the common nuclear transcription.

## Evidence of lifetime adaptation of mitochondria

There is considerably more evidence for negative natural selection of damaged mitochondria than for positive replicative adaption of variants, partly because the former involves removal of aberrant genetic variations. For example, Sharpley et al. (2012) showed that tissue-specific selection happens in heteroplasmic mice and that tissue-specific failure to select for a specific mtDNA haplotype may be detrimental, when the tissue is exposed to insufficient energy supply (Lane, 2012; Sharpley et al., 2012). Importantly, tissue-specific selection may also depend on the nuclear genome. Thus, Mootha et al. (2003) showed that mitochondria may differ by as much as 50% of their proteome content in different cells (Mootha et al., 2003).

Single-cell studies show that aged cells tend to possess only a single type of mtDNA mutation, which can differ among cells. Mitochondria with such single mtDNA mutation replace wild type (wt) mitochondria to a large degree (Khrapko et al., 1999; Cao et al., 2001), as an indication of selective clonal expansion of individual mtDNA molecules inside the network of fused mitochondria (Kowald and Kirkwood, 2011b). Yet, the lack of multiple mutations in single aged mitochondria remains indicative of some selective elimination of inefficient mitochondria by mitophagy. Imposing a more fatty acid intensive metabolism on the organism affects the biogenesis of mitochondria (Lopez-Lluch et al., 2008), as evident in the biogenetic response to hibernation, which ensures that hibernating animals have more abundant mitochondria and hence do not lose muscle mass (Harlow et al., 2001; Xu et al., 2013). The biogenetic response suggests that different environmental challenges such as limited carbohydrate availability alter the function of mitochondria and therefore potentially change the selection pressure.

Findings from bacteria show that clonal populations may exhibit cell-to-cell variation in response to stress. Very recent findings show that variability persists through cell division events, and that epigenetic inheritance contributes to the propagation of the observed phenotypic variation (Ni et al., 2012). Since mitochondria are believed to be of bacterial origin, it is likely that natural selection of the optimally adapted mitochondria involves similar epigenetic as well as genetic inheritance.

Studies on mtDNA mutator knock-in mice, a model for premature aging due to somatic mitochondrial damages, show that 5 months of endurance exercise rescues the progeroid phenotype and induces dramatic systemic mitochondrial rejuvenation, including fewer mitochondria with damaged DNA (Safdar et al., 2011). Such an exercise scheme may be regarded as a stress-induced augmentation of the natural selection of the optimally adapted mitochondria. Either the defective proof reading gene of the mtDNA mutator mice did not introduce many errors, or the mutations that did happen failed to expand clonally and were selectively removed. In either case, the study built a strong case for stress- and recovery-induced rejuvenation in this model of aging. The dramatic effects observed in this study confirm previous findings (Wright et al., 2004) that divergent biological phenomena have convergent pathways that are linked to aging, and that manipulation of stress response mechanisms have the potential for multisystem disease protection.

## Conclusions

This perspective deals with the notion that adaptive stress responses to respiratory challenges and stimulation drive natural selection of genetically and epigenetically inherited properties of mitochondria:

- When brain energy turnover increasingly depends on ketone body or fatty acid metabolism rather than on glucose, sparing of complex I and proliferation of mitochondria is beneficial to overall mitochondrial health.
- High glucose availability for oxidative phosphorylation, on the other hand, establishes a state of low selection pressure with increased accumulation of lesions.
- Intermittent non-chronic insults with increased ROS production benefit mitochondrial health and promote healthy aging and increased longevity.
- In healthy tissue, transient non-lethal insults such as chemotherapy, hypoglycemia, or hypoxic challenges, select mitochondria that are more resilient to subsequent challenges. These mitochondria are better adapted and more numerous.
- Stressful challenges with increased ROS levels, followed by subsequent recovery and treatment with biogenesis-promoting agents, yield mitochondria with greater respiratory capacity than mitochondria treated with biogenesis-promoting agents alone.

These claims have specific and testable implications, the resolution of which can revise the general understanding of the role of mitochondrial challenges in healthy aging.

### Conflict of interest statement

The authors declare that the research was conducted in the absence of any commercial or financial relationships that could be construed as a potential conflict of interest.

## References

[B1] Adam M. Murali B. Glenn N. O. Potter S. S. (2008). Epigenetic inheritance based evolution of antibiotic resistance in bacteria. BMC Evol. Biol. 8:52 10.1186/1471-2148-8-52 18282299 PMC2262874

[B2] Bergersen L. Waerhaug O. Helm J. Thomas M. Laake P. Davies A. J. (2001). A novel postsynaptic density protein: the monocarboxylate transporter MCT2 is co-localized with delta-glutamate receptors in postsynaptic densities of parallel fiber-Purkinje cell synapses. Exp. Brain. Res. 136, 523–534 10.1007/s002210000600 11291733

[B3] Bergersen L. H. Gjedde A. (2012). Is lactate a volume transmitter of metabolic states of the brain? Front. Neuroenergetics 4:5 10.3389/fnene.2012.00005 22457647 PMC3307048

[B4] Blackstone N. W. Green D. R. (1999). The evolution of a mechanism of cell suicide. Bioessays 21, 84–88 10070258 10.1002/(SICI)1521-1878(199901)21:1<84::AID-BIES11>3.0.CO;2-0

[B5] Bolaños J. P. Almeida A. Moncada S. (2009). Glycolysis: a bioenergetic or a survival pathway? Trends Biochem. Sci. 35, 145–149 20006513 10.1016/j.tibs.2009.10.006

[B6] Cairns C. B. Walther J. Harken A. H. Banerjee A. (1998). Mitochondrial oxidative phosphorylation thermodynamic efficiencies reflect physiological organ roles. Am. J. Physiol. 274(5 Pt 2), R1376–R1383 9612405 10.1152/ajpregu.1998.274.5.R1376

[B7] Cao Z. Wanagat J. McKiernan S. H. Aiken J. M. (2001). Mitochondrial DNA deletion mutations are con-comitant with ragged red regions of individual, aged muscle fibers: analysis by laser-capture microdissec-tion. Nucl. Acids Res. 29, 4502–4508 10.1093/nar/29.21.4502 11691938 PMC60181

[B8] Chau M. D. Gao J. Yang Q. Wu Z. Gromada J. (2010). Fibroblast growth factor 21 regulates energy metabolism by activating the AMPK-SIRT1-PGC-1alpha pathway. Proc. Natl. Acad. Sci. U.S.A. 107, 12553–12558 10.1073/pnas.1006962107 20616029 PMC2906565

[B9] Dai Y. Kiselak T. Clark J. Clore E. Zheng K. Cheng A. (2013). Behavioral and metabolic characterization of heterozygous and homozygous POLG mutator mice. Mitochondrion 13, 282–291 10.1016/j.mito.2013.03.006 23542163 PMC3682692

[B10] Davies K. J. Quintanilha A. T. Brooks G. A. Packer L. (1982). Free radicals and tissue damage produced by exercise. Biochem. Biophys. Res. Commun. 107, 1198–1205 10.1016/S0006-291X(82)80124-1 6291524

[B11] Dresler C. Hansen T. L. Frederiksen J. B. Marcker M. L. Singh K. K. Juel Rasmussen L. (2012). Is there a link between mitochondrial reserve respiratory capacity and aging? J. Aging Res. 2012:192503 22720157 10.1155/2012/192503 PMC3375017

[B11a] DuBoff B. Feany M. Götz J. (2013). Why size matters - balancing mitochondrial dynamics in Alzheimer's disease. Trends Neurosci. 36, 325–335 10.1016/j.tins.2013.03.002 23582339

[B12] Duvezin-Caubet S. Jagasia R. Wagener J. Hofmann S. Trifunovic A. Hansson A. (2006). Proteolytic processing of OPA1 links mitochondrial dysfunction to alterations in mitochondrial morphology. J. Biol. Chem. 281:379729 10.1074/jbc.M606059200 17003040

[B12a] Edelman G. M. Mountcastle V. B. (1978). The Mindful Brain, New York, NY: Plenum Press

[B12b] Edelman G. M. Reeke G. N. Jr. (1982). Selective networks capable of representative transformations, limited generalizations, and associative memory. Proc. Natl. Acad. Sci. U.S.A. 79, 2091–2095 10.1073/pnas.79.6.2091 6952255 PMC346129

[B13] Elliott H. R. Samuels D. C. Eden J. A. Relton C. L. Chinnery P. F. (2008). Pathogenic mitochondrial DNA mutations are common in the general population. Am. J. Hum. Genet. 83, 254–260 10.1016/j.ajhg.2008.07.004 18674747 PMC2495064

[B14] Enríquez J. A. Fernández-Sílva P. Montoya J. (1999). Autonomous regulation in mammalian mitochondrial DNA transcription. Biol. Chem. 380, 737–747 10.1515/BC.1999.094 10494823

[B15] Feng D. Liu L. Zhu Y. Chen Q. (2013). Molecular signaling toward mitophagy and its physiological significance. Exp. Cell Res. pii, S0014-482700159-6. 23603281 10.1016/j.yexcr.2013.03.034

[B16] Giles R. E. Blanc H. Cann H. M. Wallace D. C. (1980). Maternal inheritance of human mitochondrial DNA. Proc. Natl. Acad. Sci. U.S.A. 77, 6715–6719 10.1073/pnas.77.11.6715 6256757 PMC350359

[B17] Gjedde A. Crone C. (1975). Induction processes in blood-brain transfer of ketone bodies during starvation. Am. J. Physiol. 229, 1165–1169 1200135 10.1152/ajplegacy.1975.229.5.1165

[B18] Gjedde A. Johannsen P. Cold G. E. Ostergaard L. (2005). Cerebral metabolic response to low blood flow: possible role of cytochrome oxidase inhibition. J. Cereb. Blood Flow Metab. 25, 1183–1196 10.1038/sj.jcbfm.9600113 15815583

[B19] Grossniklaus U. Kelly B. Ferguson-Smith A. C. Pembrey M. Lindquist S. (2013). Transgenerational epigenetic inheritance: how important is it? Nat. Rev. Genet. 14, 228–235 10.1038/nrg3435 23416892 PMC4066847

[B20] Gurd B. J. Yoshida Y. McFarlan J. T. Holloway G. P. Moyes C. D. Heigenhauser G. J. (2011). Nuclear SIRT1 activity, but not protein content, regulates mitochondrial biogenesis in rat and human skeletal muscle. Am. J. Physiol. Regul. Integr. Comp. Physiol. 301, R67–R75 10.1152/ajpregu.00417.2010 21543634

[B21] Harlow H. J. Lohuis T. Beck T. D. Iaizzo P. A. (2001). Muscle strength in overwintering bears. Nature 409, 997 10.1038/35059165 11234052

[B22] Hengartner M. O. (1998). Apoptosis. Death cycle and Swiss army knives. Nature 391, 441–442 10.1038/35036 9461208

[B22a] He Y. Wu J. Dressman D. C. Iacobuzio-Donahue C. Markowitz S. D. Velculescu V. E. (2010). Heteroplasmic mitochondrial DNA mutations in normal and tumour cells. Nature 464, 610–614 10.1038/nature08802 20200521 PMC3176451

[B23] Jansen R. P. de Boer K. (1998). The bottleneck: mitochondrial imperatives in oogenesis and ovarian follicular fate. Mol. Cell. Endocrinol. 145, 81–88 10.1016/S0303-7207(98)00173-7 9922103

[B24] Jerne N. K. (1955). The natural-selection theory of antibody formation. Proc. Natl. Acad. Sci. U.S.A. 41, 849–857 10.1073/pnas.41.11.849 16589759 PMC534292

[B25] Jose C. Melser S. Benard G. Rossignol R. (2013). Mitoplasticity: adaptation biology of the mitochondrion to the cellular redox state in physiology and carcinogenesis. Antioxid Redox Signal. 18, 808–849 10.1089/ars.2011.4357 22989324

[B26] Kang H. C. Lee Y. M. Kim H. D. (2013). Mitochondrial disease and epilepsy. Brain Dev. pii, S0387–S7604. 10.1016/j.braindev.2013.01.006 23414619

[B27] Khrapko K. Bodyak N. Thilly W. G. van Orsouw N. J. Zhang X. Coller H. A. (1999). Cell by cell scanning of whole mitochondrial genomes in aged human heart reveals a significant fraction of myocytes with clonally expanded deletions. Nucl. Acids Res. 27, 2434–2341 10.1093/nar/27.11.2434 10325435 PMC148812

[B28] Knott A. B. Bossy-Wetzel E. (2008). Impairing the mitochondrial fission and fusion balance: a new mechanism of neurodegeneration. Ann. N.Y. Acad. Sci. 1147, 283–292 10.1196/annals.1427.030 19076450 PMC2605288

[B29] Kowald A. Jendrach M. Pohl S. Bereiter-Hahn J. Hammerstein P. (2005). On the relevance of mitochondrial fusions for the accumulation of mitochondrial deletion mutants: a modelling study. Aging Cell 4, 273–283 10.1111/j.1474-9726.2005.00169.x 16164426

[B30] Kowald A. Kirkwood T. B. (2011a). Evolution of the mitochondrial fusionfission cycle and its role in aging. Proc. Natl. Acad. Sci. U.S.A. 108, 1023742 Erratum in: *Proc. Natl. Acad. Sci. U.S.A.* 108, 16481. 10.1073/pnas.1101604108 21646529 PMC3121810

[B31] Kowald A. Kirkwood T. B. (2011b). The evolution and role of mitochondrial fusion and fission in aging and disease. Commun. Integr. Biol. 4, 6279 22046482 10.4161/cib.4.5.17110 PMC3204148

[B32] Kuznetsov A. V. Margreiter R. (2009). Heterogeneity of mitochondria and mitochondrial function within cells as another level of mitochondrial complexity. Int. J. Mol. Sci. 10, 1911–1929 10.3390/ijms10041911 19468346 PMC2680654

[B33] Lane N. (2011). Mitonuclear match: otimizing fitness and tertility over generations drives ageing wtihing generations. Bioessays 33, 860–869 21922504 10.1002/bies.201100051

[B34] Lane N. (2012). The problem with mixing mitochondria. Cell 151, 246–248 10.1016/j.cell.2012.09.028 23063117

[B35] Lauritzen K. H. Morland C. Puchades M. Holm-Hansen S. Hagelin E. M. Lauritzen F. (2013). Lactate receptor sites link neurotransmission, neurovascular coupling and brain energy metabolism. Cereb, Cortex [Accepted 15th of April]. [Epub ahead of print]. 23696276 10.1093/cercor/bht136

[B36] Li Z. Okamoto K. Hayashi Y. Sheng M. (2004). The importance of dendritic mitochondria in the morphogenesis and plasticity of spines and synapses. Cell 119, 873–887 10.1016/j.cell.2004.11.003 15607982

[B37] Lopez-Lluch G. Irusta P. M. Navas P. de Cabo R. (2008). Mitochondrial biogenesis and healthy aging. Exp. Gerontol. 43, 813–819 10.1016/j.exger.2008.06.014 18662766 PMC2562606

[B38] Minocherhomji S. Tollefsbol T. O. Singh K. K. (2012). Mitochondrial regulation of epigenetics and its role in human diseases. Epigenetics 7, 326–334 10.4161/epi.19547 22419065 PMC3368816

[B39] Mootha V. K. Bunkenborg J. Olsen J. V. Hjerrild M. Wisniewski J. R. Stahl E. (2003). Integrated analysis of protein composition, tissue diversity, and gene regulation in mouse mitochondria. Cell 115, 629–640 10.1016/S0092-8674(03)00926-7 14651853

[B40] Ni M. Decrulle A. L. Fontaine F. Demarez A. Taddei F. Lindner A. B. (2012). Pre-disposition and epigenetics govern variation in bacterial survival upon stress. PLoS Genet. 8:e1003148 10.1371/journal.pgen.1003148 23284305 PMC3527273

[B41] Payne B. A. Wilson I. J. Yu-Wai-Man P. Coxhead J. Deehan D. Horvath R. (2013). Universal heteroplasmy of human mitochondrial DNA. Hum. Mol. Genet. 22, 384–390 10.1093/hmg/dds435 23077218 PMC3526165

[B42] Quistorff B. Secher N. H. Van Lieshout J. J. (2008). Lactate fuels the human brain during exercise. FASEB J. 22, 3443–3449 10.1096/fj.08-106104 18653766

[B43] Radak Z. Zhao Z. Koltai E. Ohno H. Atalay M. (2013). Oxygen consumption and usage during physical exercise: the balance between oxidative stress and ROS-dependent adaptive signaling. Antiox. Redox Signal. 18, 1208–1246 10.1089/ars.2011.4498 22978553 PMC3579386

[B44] Rasmussen P. Nielsen J. Overgaard M. Krogh-Madsen R. Gjedde A. Secher N. H. (2010a). Reduced muscle activation during exercise related to brain oxygenation and metabolism in humans. J. Physiol. 588(Pt 11), 1985–1995 20403976 10.1113/jphysiol.2009.186767 PMC2901984

[B45] Rasmussen P. Nybo L. Volianitis S. Møller K. Secher N. H. Gjedde A. (2010b). Cerebral oxygenation is reduced during hyperthermic exercise in humans. Acta Physiol. (Oxf). 199, 63–70 10.1111/j.1748-1716.2010.02084.x 20102344

[B46] Safdar A. Bourgeois J. M. Ogborn D. I. Little J. P. Hettinga B. P. Akhtar M. (2011). Endurance exercise rescues progeroid aging and induces systemic mitochondrial rejuvenation in mtDNA mutator mice. Proc. Natl. Acad. Sci. U.S.A. 108, 4135–4140 10.1073/pnas.1019581108 21368114 PMC3053975

[B47] Sharpley M. S. Marciniak C. Eckel-Mahan K. McManus M. Crimi M. Waymire K. (2012). Heteroplasmy of mouse mtDNA is genetically unstable and results in altered behavior and cognition. Cell 151, 333–343 10.1016/j.cell.2012.09.004 23063123 PMC4175720

[B48] Seifert T. S. Brassard P. Jorgensen T. B. Hamada A. J. Rasmussen P. Quistorff B. (2009). Cerebral non-oxidative carbohydrate consumption in humans driven by adrenaline. J. Physiol. 587, 285–293 10.1113/jphysiol.2008.162073 19015195 PMC2670041

[B49] Taylor R. W. Turnbull D. M. (2005). Mitochondrial DNA mutations in human disease. Nat. Rev. Genet. 6, 389–402 10.1038/nrg1606 15861210 PMC1762815

[B50] Twig G. Elorza A. Molina A. J. Mohamed H. Wikstrom J. D. Walzer G. (2008). Fission and selective fusion govern mitochondrial segregation and elimination by autophagy. EMBO J. 27, 43346 10.1038/sj.emboj.7601963 18200046 PMC2234339

[B51] Twig G. Shirihai O. S. (2011). The interplay between mitochondrial dynamics and mitophagy. Antioxid Redox Signal. 14, 1939–1951 10.1089/ars.2010.3779 21128700 PMC3078508

[B52] Vafaee M. S. Vang K. Bergersen L. H. Gjedde A. (2012). Oxygen consumption and blood flow coupling in human motor cortex during intense finger tapping: implication for a role of lactate. J. Cereb. Blood Flow Metab. 32, 1859–1868 10.1038/jcbfm.2012.89 22781333 PMC3463880

[B53] Wright A. F. Jacobson S. G. Cideciyan A. V. Roman A. J. Shu X. Vlachantoni D. (2004). Lifespan and mitochondrial control of neurodegeneration. Nat. Genet. 36, 11538 10.1038/ng1448 15514669

[B54] Xu R. Andres-Mateos E. Mejias R. Macdonald E. M. Leinwand L. A. Merriman D. K. (2013). Hibernating squirrel muscle activates the endurance exercise pathway despite prolonged immobilization. Exp. Neurol. pii, S0014-4886000186. 23333568 10.1016/j.expneurol.2013.01.005 PMC3706566

[B55] Zhang Y. Kuang Y. Xu K. Harris D. Lee Z. Lamanna J. (2013). Ketosis proportionately spares glucose utilization in brain. J. Cereb. Blood Flow Metab. [Epub ahead of print]. 10.1038/jcbfm.2013.87 23736643 PMC3734783

